# Closed Fluxtubes and Dispersive Proton Conics at Jupiter's Polar Cap

**DOI:** 10.1029/2022GL098741

**Published:** 2022-04-29

**Authors:** J. R. Szalay, G. Clark, G. Livadiotis, D. J. McComas, D. G. Mitchell, J. S. Rankin, A. H. Sulaiman, F. Allegrini, F. Bagenal, R. W. Ebert, G. R. Gladstone, W. S. Kurth, B. H. Mauk, P. W. Valek, R. J. Wilson, S. J. Bolton

**Affiliations:** ^1^ Department of Astrophysical Sciences Princeton University Princeton NJ USA; ^2^ The Johns Hopkins University Applied Physics Laboratory Laurel MD USA; ^3^ University of Iowa Iowa IA USA; ^4^ Southwest Research Institute San Antonio TX USA; ^5^ Department of Physics and Astronomy University of Texas at San Antonio San Antonio TX USA; ^6^ Laboratory for Atmospheric and Space Physics University of Colorado Boulder Boulder CO USA

## Abstract

Two distinct proton populations are observed over Jupiter's southern polar cap: a ∼1 keV core population and ∼1–300 keV dispersive conic population at 6–7 R_J_ planetocentric distance. We find the 1 keV core protons are likely the seed population for the higher‐energy dispersive conics, which are accelerated from a distance of ∼3–5 R_J_. Transient wave‐particle heating in a “pressure‐cooker” process is likely responsible for this proton acceleration. The plasma characteristics and composition during this period show Jupiter's polar‐most field lines can be topologically closed, with conjugate magnetic footpoints connected to both hemispheres. Finally, these observations demonstrate energetic protons can be accelerated into Jupiter's magnetotail via wave‐particle coupling.

## Key Points

- Dispersive proton conics are observed connected to Jupiter's polar cap
- Inferring perpendicular heating, the source region for these events is within a jovicentric distance of ∼3–5 R_J_
- Plasma composition and trapped protons indicate closed magnetic structures exists even at Jupiter's most polar regions

## 1 Introduction

The polar regions of Jupiter's magnetosphere are unlike those of any other planet explored by spacecraft with an internal magnetic field. Mercury (e.g., Anderson et al., 2010), Earth (e.g., Smith & Lockwood, 1996), and Saturn (e.g., Jasinski et al., 2014) all exhibit open magnetic topologies in their polar‐most regions, where the solar wind is magnetically connected to each of these planets. Evidence for this solar wind connectivity has been found in magnetic field signatures associated with magnetopause transits (e.g., Gurnett et al., 2010) and direct observation of solar wind plasma composition and polar rain – field aligned solar wind strahl electrons that stream along the interconnected magnetic field toward the planet (Winningham & Heikkila, 1974).

**Figure 1:**
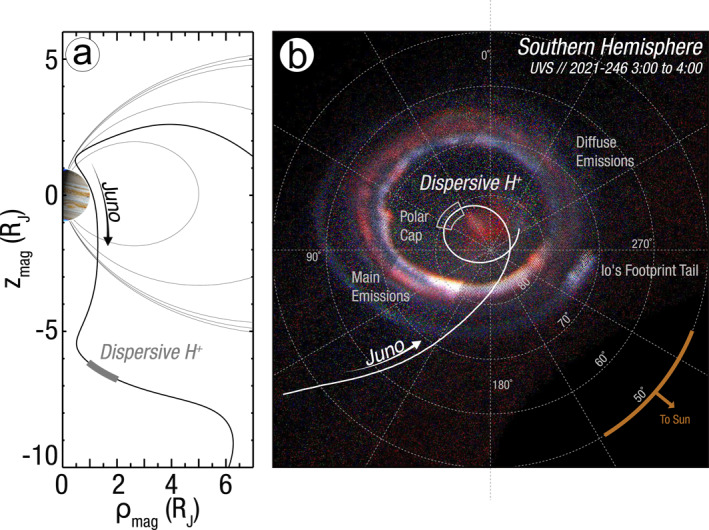
(a) The Juno trajectory in a coordinate system with the *z*‐axis aligned with Jupiter's magnetic dipole. (b) The Juno footprint mapped to Jupiter using magnetic field models along with ultraviolet images of the auroral emissions, with labels for the distinct regions and solar direction. The grey boxes indicate where Juno observed dispersive proton conics.

Jupiter's magnetosphere is internally driven by its strong, rapidly rotating magnetic field and Io's immense heavy ion plasma source. It likely has a fundamentally different magnetospheric interaction with the solar wind than the aforementioned planets (McComas & Bagenal, 2007). Jupiter's intense auroral features are primarily driven by plasma processes throughout the magnetosphere and are an important diagnostic in understanding these complex processes (Delamere & Bagenal, 2010). These auroral features (e.g., Grodent, 2016) are typically categorized into: the oval‐shaped main auroral emission, Galilean satellite footprint aurora, diffuse emissions equatorward of the main oval, and polar cap emissions poleward of the main oval (Figure 1). Polar cap emissions can exhibit rapid variations (e.g., Bonfond et al., 2016) and the mechanisms sustaining these emissions remain a mystery (e.g., Ebert et al., 2019). As the most poleward auroral features would map to the farthest distances from Jupiter, these auroral emissions have been used to diagnose Jupiter's solar wind interaction. Polar cap emissions have previously been interpreted to infer the presence of a Jovian cusp (Bunce et al., 2004). In contrast, a subsequent analysis interpreted these emissions to indicate a highly complex topological setup where the large majority of Jupiter's polar cap is threaded by closed magnetic fields (Zhang et al., 2021). One of primary goals of the Juno mission (Bolton et al., 2017) is to use its polar orbit to characterize Jupiter's high latitude magnetosphere and assess its magnetic topology (Bagenal et al., 2017).

Since arriving at Jupiter, Juno has directly sampled the fields and particles connected to the polar cap, while also collecting infrared and ultraviolet (UV) images of these regions. When magnetically connected equatorward of the polar caps, Juno observed diverse ion and electron populations associated with both ionospheric and Io‐genic plasma sources (Allegrini et al., 2017; Clark et al., 2018; Mauk et al., 2017, 2018; Szalay et al., 2017, 2021; Valek et al., 2019). Energetic ion conics, likely due to perpendicular heating via wave‐particle coupling, were observed just poleward of the main emissions (Clark, Mauk, Paranicas, et al., 2017). When connected to the polar cap, ion and electron populations over large energy ranges exhibit both broadband and electrostatic acceleration (Clark, Mauk, Haggerty, et al., 2017; Mauk et al., 2020). Whistler‐mode waves were determined to interact with electrons via pitch angle scattering (Elliott et al., 2018) and be generated by electron beams (Elliott et al., 2020). These regions have been observed to be mostly devoid of low energy plasma at very low altitudes (Pollock et al., 2020). Outward fluxes along polar magnetic fields of low energy ∼10's keV electrons (Ebert et al., 2017) and high energy ∼1 MeV electrons (Clark et al., 2020) appear to be a common feature within Jupiter's polar cap. While acceleration via magnetic reconnection has been proposed as a source mechanism for outward electrons (Masters et al., 2021), direct evidence of this reconnection has not been observed in‐situ. The exact origins and source mechanisms sustaining polar cap electron populations and the temporal variability of their auroral emissions remain a mystery.

There are still fundamental and unresolved questions relating to the origins of Jupiter's polar cap and its relation to Jupiter's solar wind interaction: (a) What are the acceleration mechanisms operating in the polar cap and what drives fast temporal variations? (b) Are the polar caps magnetically connected to the solar wind at all and if so, how and to what extent? (c) What processes inject charged particles into Jupiter's magnetotail? Juno is uniquely suited to address these questions with its comprehensive suite of in‐situ and remote‐sensing instrumentation. Here, we investigate polar fields, particle, and UV imaging observations from JADE – the Jovian Auroral Distributions Experiment (McComas, Alexander, et al., 2017), JEDI – the Jupiter Energetic particle Detector Instrument (Mauk et al., 2017), the Waves instrument (Kurth et al., 2017), and UVS – the UV Spectrograph (Gladstone et al., 2017) onboard the Juno spacecraft (Bolton et al., 2017). In Section 2, we describe distinct accelerated proton events observed to arrive with time‐energy dispersion and estimate their source regions. We highlight how these observations provide evidence for closed magnetic topology during this period in Section 3 and conclude with a discussion on the results and implications in Section 4.

## 2 Dispersive Proton Events

Juno's high inclination orbit in the latter phase of the mission is oriented such that it traverses the highest latitudes of the southern polar cap region. Figure 1a shows the Juno orbit, where the dispersive events examined in this work are observed within 2 R_J_ cylindrical radial distance from Jupiter's magnetic dipole axis (1 R_J_ ≡ 71,492 km). Figure 1b shows a composite UV image from the UVS instrument, where overall brightness encodes emission intensity and red indicates lower altitude emissions (more energetic precipitating electron fluxes) while white indicates higher altitude emissions (less energetic precipitation). Overlaid on this image is the Juno footprint in the southern hemisphere, traced back from Juno's position to Jupiter using the JRM09 internal field model (Connerney et al., 2018) and current sheet model (Connerney et al., 2020). As shown here, the dispersive events observed by Juno map to a System III longitude of 40°–60° and latitude of 80°, well within the polar cap, at 23:00–24:00 local time. Both panels in Figure 1 highlight the period during Juno's 36th periojove of 2021‐246 3:00–3:50 UTC when dispersive proton features are observed. The UV image was integrated for an additional 10 min for improved statistics.

**Figure 2:**
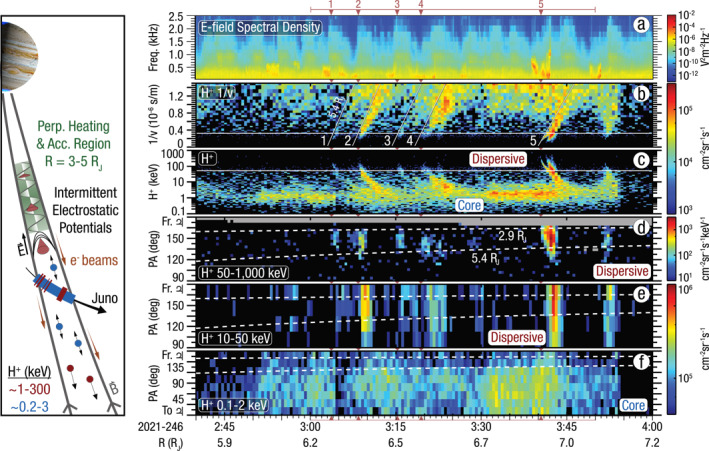
Waves and particle data as a function of time with all five dispersive proton features marked with red triangles. Panel (a) shows the electric field spectral density. Panels (b) and (c) show H^+^ flux as a function of inverse speed and energy respectively. Lines in panel (b) show a dispersion relation for ions perpendicularly heated at 5.4 R_J_ planetocentric distance. Panels (d), (e), and (f) show H^+^ flux as a function of pitch angle (PA) for 50 to 1,000 keV, 10–50 keV and 0.1–2 keV, respectively. Each pixel in the Jovian Auroral Distributions Experiment (JADE) PA spectrograms (e) and (f) are 22.5° wide, which is the resolution of an individual JADE‐I anode. Horizontal dashed lines in panels (d–f) show expected PAs for perpendicularly heated ions originating at 2.9 R_J_ and 5.4 R_J_. A schematic highlighting the acceleration mechanism is shown on the left.

Two proton populations are observed across JADE and JEDI, shown in Figures 2b and 2c. A “core” low‐energy population centered around 1 keV is initially weakly present with growing intensities and is nearly isotropic (Figure 2f). This population abruptly disappears at 3:54 indicating Juno transited to flux tubes depleted in protons. No appreciable fluxes are observed below ∼0.2 keV. A second population of protons exhibiting a dispersive time‐energy profile is intermittently observed from ∼3:00–3:50 (marked with red lines) at energies above the core ∼1 keV population up to 100's of keV, where at least five events exhibit distinct features with higher energy protons followed by lower energy protons. A sixth event ∼3:51 also appears to be dispersive, but is less distinguishable than the five identified events.

Diagonal features in energy‐time spectrograms are indicative of velocity dispersion, where particles accelerated over a range of energies at similar start times and locations arrive with delays depending on particle speed. To assess the nature of the dispersion characteristics, we display the fluxes as a function of inverse velocity in panel (b).

In panel (d), the highest energy fluxes observed first for each event occur at PAs offset from perfectly field aligned, traveling away from Jupiter with a conic PA distribution. As conics are typically caused by perpendicular heating and outward motion due to the magnetic mirror force, we can estimate their source regions with four assumptions: (a) ions are initially perpendicularly heated and originate with PA = 90°; (b) they accelerate away from Jupiter via the magnetic mirror force conserving the first adiabatic invariant; (c) no appreciable acceleration occurs between the perpendicular heating region and Juno; and (d) the magnetic field is dipolar in these regions. With these assumptions, the acceleration region distance is $r_{source}=r_{s/c}{\left(\sin\;\alpha_{s/c}\right)}^{2/3}$, where *r*_*s/c*_ is Juno's planetocentric distance and *α*_*s/c*_ is the PA observed at Juno. The full width half maximum at the onset of each event for fluxes above 50 keV is: (a) 136°–159°, (b) 132°–154°, (c) 133°–156°, (d) 130°–151°, and (e) 148°–164°. From these PAs, we estimate the source region exists in the range of 2.9 R_J_ to 5.4 R_J_. Figure 2 shows the expected PAs for ions originating at these distances in panels (d)‐(f) as dashed white lines.

Using the higher limit of 5.4 R_J_, we show the expected dispersion relation for conics with diagonal lines in Figure 2b. All events exhibit a slope in 1/v space near or less than that for a source at 5.4 R_J_. Hence the derived source regions from the conic analysis and the slope of the dispersion relation are both consistent with a source region below 5.4 R_J_. We note the strongest event 5 is composed of two closely timed dispersion features that almost overlap. In the range of dispersion event onset times, Juno has the following parameters: distance of 6.3–6.9 R_J_, perpendicular distance from Jupiter's magnetic dipole of 1.9–2.2 R_J_, relative speed with respect to Jupiter of 22–23 km s^−1^ corresponding to an offset energy of 2–3 eV, and local corotation speed of 24–28 km s^−1^ corresponding to a proton energy of 3–4 eV. With low energies relative to even the core ∼1 keV proton population, the spacecraft motion and local corotation are negligible in calculating for the dispersion timing and plasma‐frame energy.

The core population of ∼1 keV protons present during this period provides critical context for the dispersive conics. It is nearly isotropic with respect to the local magnetic field direction, with the exception of a PA deficiency above 135° coming from Jupiter (Figure 2f). If there were no acceleration processes operating, nearly equal fluxes in the 0°–45° range as in the 135°–180° should be present, since the upgoing protons outside the loss cone of ∼3° would mirror back away from the planet unperturbed. The PA deficiency indicates a reservoir of low energy protons that would otherwise mirror away from the planet have been accelerated. JADE resolves the onset of pitch angle deficiency within 135°–157.5°. Assuming adiabatic conservation of the magnetic moment as before, protons moving toward Jupiter with pitch angles from 22.5° to 45° during this period would mirror within a distance of 3.3–5.6 R_J_. Hence, the range of PAs associated with the dropout of low energy protons is consistent with the peak conic PAs. Therefore, we suggest the low energy core protons are the seed population for the accelerated dispersive protons.

Due to the spacecraft orientation, magnetic field geometry, and instrument mounting, neither JADE nor JEDI was able to fully observe field aligned electrons during this period. However, the Waves instrument provides independent insight about electron characteristics. Figure 2a shows the electric field spectral density recorded by the Waves instrument in a frequency‐time spectrogram. Coincident with the dispersion events are plasma wave emissions that exhibit a dispersive spectral character, most pronounced for Event 5 whereby higher frequencies are detected farther from the source magnetic field line. The whistler mode's theoretical upper frequency cutoff is at the electron cyclotron, *f*_*ce*_, or electron plasma frequency, *f*_*pe*_, whichever is lower. During this interval, *f*_*ce*_ was 9.9–6.2 kHz and the upper frequency cutoff of Event 5 at ∼1.3 kHz was in good agreement with *f*_*pe*_ ∼ 1.6 kHz, for an estimated JADE H^+^ numerical density of 0.03 cm^−3^ and assuming quasi‐neutrality. The quasi‐electrostatic whistler mode does not propagate below the lower hybrid frequency, which approximates the ion plasma frequency, *f*_*pi*_, for a mildly under‐dense plasma as such. The lack of a lower frequency cutoff further supports this mode as the emission is observed down to the 50 Hz lower frequency limit of Waves, which is above the lower hybrid frequency of ∼38 Hz. Finally, the calculated *E*/*cB* (not shown here) is ∼10, confirming a quasi‐electrostatic mode. Note, the frequency dispersion is not directly linked to the observed energy dispersion of the ions.

Altogether, these emissions are consistent with the whistler‐mode propagating near the resonance cone, often called auroral hiss (e.g., Sulaiman et al., 2018). Their generation mechanism is via a beam‐plasma instability when the Landau resonance condition is met, that is, the parallel phase velocity matches the beam velocity (e.g., Farrell et al., 1988; James, 1976). They are therefore commonly observed in the presence of electron beams and have been used as reliable indicators of auroral particle acceleration (e.g., Ergun et al., 2003; Kopf et al., 2010). The Landau resonance condition requires that the waves and electrons travel in the same direction. The fine structures of the plasma wave emissions, particularly for Event 5, strongly suggest the waves originate from a source below Juno where the acceleration region is estimated to be. If these emissions were originating from farther out in the magnetosphere, we would expect the waves to be much fainter or completely damped. We therefore conclude that magnetic field‐aligned electron beams are propagating upward as the source of free energy for the generation of the observed whistler‐mode auroral hiss.

## 3 Open vs. Closed Magnetospheric Configuration

In addition to exposing acceleration mechanisms operating in Jupiter's polar cap, these observations provide an opportunity to investigate whether Juno is on open or closed magnetic structures. At Earth's open magnetic regions, plasma populations are significantly depleted on open field lines, where typically only strahl electrons streaming in field‐aligned directions toward the planet are observed (e.g., Winningham & Heikkila, 1974). Here, while we are unable to assess the existence of strahl electrons due to lack of electron PA coverage, we observe a core proton population with a nearly isotropic PA distribution. This shows approximately half the protons are traveling towards Jupiter, indicating Juno was connected to magnetic field lines already loaded with protons for some time, in contrast to the nominal empty terrestrial open polar counterpart.

**Figure 3:**
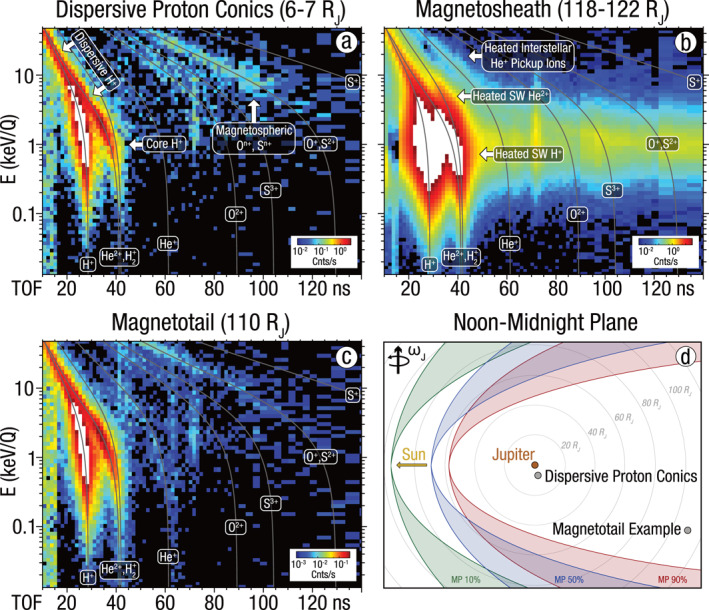
Time‐of‐flight count rates observed by the Jovian Auroral Distributions Experiment as a function of mass per charge and energy per charge for (a) the dispersive conic events, (b) the magnetosheath, and (c) the magnetotail. The core and dispersive proton populations are marked in (a), where the lower count rate “fork” is an instrumental feature also due to protons. During this time, magnetospheric heavy ions (O^n+^, S^n+^) are observed above ∼5 keV, exhibiting significant similarities to the composition of the magnetotail. Panel (d) shows the magnetosphere in the noon‐midnight plane, with the locations of (a) and (c) and three ranges of magnetopause boundaries (Ranquist et al., 2020).

Separately, the plasma composition gives critical insight on the connectivity. Figure 3a shows JADE count rates as a function of mass per charge and energy per charge from 2021‐246 3:00 to 3:50. The two proton populations are evident in this observation near the M/Q = 1 line, which splits into a “fork” because incoming protons transit through a carbon foil in the instrument and exit with approximately 80% as neutral H in the lower TOF fork and 20% as H^+^ in the higher TOF fork (Kim et al., 2020; McComas, Alexander, et al., 2017).

Figure 3b shows a TOF spectra when Juno transited the dawnside magnetosheath on its initial approach to Jupiter (McComas, Szalay, et al., 2017) from 2016‐077 0:00 to 12:00. As shown here, the plasma composition is significantly different from the proton conic events. There are three identifiable populations in the sheath plasma: (a) heated solar wind protons ∼1 keV, (b) heated solar wind He^2+^, and (c) heated He^+^ interstellar pickup ions. Each population exhibits signatures of heating as solar wind transits Jupiter's bow shock. None of these sheath signatures are present in Figure 3a. While there are counts below 10 keV that occur near the M/Q = 2 line in Figure 3a, these are all attributable to incident protons and there is no evidence of He^2+^ present in the solar wind near Jupiter at ∼4% abundance compared to protons (e.g., Wilson et al., 2018). In contrast, as shown in Figure 3a, JADE detects appreciable count rates at M/Q = 8 and 16 corresponding to O^2+^, O^+^/S^2+^ indicative of Io‐genic Jovian plasma. Therefore, the compositions observed during these dispersive conic events are inconsistent with a magnetosheath origin.

Figure 3c shows a TOF spectra from 2020–027 to 2020–028 when Juno was deep in the magnetotail at 110 RJ, −21° Jovian latitude, and midnight local time. This event is well inside all models for the magnetopause, where Figure 3d shows the 10%, 50%, and 90% boundaries (Joy et al., 2002; Ranquist et al., 2020). This example was chosen since it was one of the farthest magnetotail locations Juno reached at similar local times to the dispersive events. The energies and composition in the magnetotail are remarkably similar to those in Figure 3a. In both time periods, a proton dominated plasma is observed with lower, yet detectable abundances of Io‐genic heavy ions. The prevalence of light ions in Jupiter's magnetotail was discovered when New Horizons transited far down the tail (McComas et al., 2007, 2017), where similarly sharp boundaries between loaded and depleted fluxtubes were observed. As the higher energy proton tail shown in Figure 3a is only present when Juno observed the dispersive proton events, Juno may have directly observed one of the processes seeding these higher energy protons (over a few keV) into the magnetotail. Note, reconnection‐driven tailward transport has been observed to occur at similar distances in the magnetotail (e.g., Delamere & Bagenal, 2013; Vogt et al., 2020) and there are still many unknowns regarding the temporal variability, local‐time, latitudinal, and radial dependence of reconnection within Jupiter's magnetotail. While we do not determine if the magnetotail plasma investigated in Figure 3c has been subject to tailward reconnection‐driven transport or not, we highlight the remarkable similarity in composition and energy distribution between tail plasma and the dispersive conic events shown in Figure 3a. In summary, the proton populations, pitch angles, and heavy ion abundances all indicate Juno was on closed magnetic field lines, connected to the magnetotail, and loaded with Jovian plasma during the period dispersive conics were observed.

## 4 Discussion and Conclusions

We present observations of dispersive proton conics connected to Jupiter's polar regions, with energies in the range of approximately 1–300 keV. A lower energy core proton population at ∼1 keV was simultaneously observed and is likely the seed population for the dispersive proton conics. Assuming perpendicular heating, we estimate the source region in three distinct ways using: (a) conic PA peak flux; (b) slope of the dispersion of high energy protons in time/inverse‐speed space; and (c) PA dropout in low‐energy core population. Each of these methods are consistent with a source region located at ∼3–5 R_J_ from Jupiter's center.

The period discussed here had the most well‐structured dispersion features observed up to and including Juno's 36th perijove. We note three other weaker potential dispersive proton features during PJ25 from 2020‐049 7:00 to 12:00, PJ30 from 2020‐313 12:00 to 18:00, and PJ32 from 2020‐052 23:00 to 2020–053 5:30. During each of these other time periods, in addition to the apparently dispersive features, a core population near 1 keV was always present. Additionally, there are many more observations of ∼1 keV protons connected to the polar cap where such dispersional proton features either do not exist or are not temporally resolved. Why the polar magnetosphere is often devoid of appreciable low energy plasma at low altitudes (Pollock et al., 2020) and what dictates the abundance of low energy plasma vs. relatively empty field lines remain an open mystery.

Multiple acceleration mechanisms have been proposed to explain auroral and plasma phenomena connected to Jupiter's polar cap. The process responsible for the accelerated dispersive features discussed here must energize protons to energies of ∼1–300 keV with a broad upward pitch angle distribution and operate on short timescales ∼10's seconds. Quasi‐static electrostatic potentials associated with Jupiter's polar regions have been observed as large as 100's keV to ∼1 MeV (Clark et al., 2017, 2020; Clark, Mauk, Haggerty, et al., 2017; Mauk et al., 2017). Near the main auroral emissions, quasi‐static potentials have also been observed to inject substantial quantities of protons into Jupiter's magnetosphere (Szalay et al., 2021), where ionospheric outflow is also required to transport ionospheric protons to these potential structures. While ionospheric outflow and some small degree of acceleration may be the source of the core ∼1 keV population, 100's keV quasistatic potentials would produce field‐aligned proton beams peaking around a characteristic energy. Here, we see acceleration across a broad range of energies and pitch angles, inconsistent with mono‐energetic quasi‐static acceleration.

Alternatively, reconnection has been proposed to exist in the polar cap (Masters et al., 2021) to explain the predominance of upward electron fluxes over downward electron fluxes (e.g., Ebert et al., 2017). Dispersive events have been observed in Jupiter's magnetotail associated with strong guide field reconnection at the magnetopause (Hill et al., 2009). However, such a process would not preferentially accelerate protons perpendicularly to produce the observed conics, and cannot explain the dispersive proton observations.

We suggest perpendicular ion‐cyclotron heating and upwelling via the outward magnetic mirror force to be the most likely explanation. At Jupiter, such a mechanism has been observed connected to its polar regions at distances of 4–6 R_J_ (Clark, Mauk, Paranicas, et al., 2017) and associated with Io with ∼1 MeV conics (Clark, Mauk, Kollmann, Szalay, et al., 2020; Sulaiman et al., 2020) likely directly connected to Io's Alfvén wing (Szalay et al., 2020). The maximum energy achievable by perpendicular heating depends on the rate waves perpendicularly accelerate protons as well as the time these protons spend within the heating region. To accelerate protons to the large energies up to ∼300 keV typically requires a “pressure‐cooker” mechanism (Gorney et al., 1985) in addition to perpendicular heating, where a quasi‐static potential traps heated ions allowing them to extract more energy from waves until they overcome the quasi‐static potential or until that structure disappears. In such a setup, the same downward electric field that pushes ions planetward will accelerate electrons anti‐planetward to form field‐aligned electron beams. In the terrestrial auroral environment, ion conics and upward electron beams have been observed in downward current regions (Carlson et al., 1998). The schematic in Figure 2 summarizes this mechanism.

The quasi‐electrostatic whistler‐mode emissions observed during this period are highly indicative of upward field‐aligned electron beams (e.g., Elliott et al., 2020), consistent with the existence of quasi‐static potential structures with planetward electric fields. Their arrival time also occurs before the proton conics, consistent with quicker electron travel. The existence and downward polarity of electric fields have been previously inferred from energetic particle data in similar polar regions (Mauk et al., 2020). Additionally, there are no enhanced emissions associated with the magnetically connected regions of the polar cap aurora during this period (Figure 2a), also consistent with an electric field accelerating electrons away from Jupiter. Therefore, outbound electron beams, presumably due to parallel potential structures, in tandem with the large energies observed for the proton conics, are consistent with pressure‐cooker ion acceleration.

Unlike dispersional solar wind features, which often exhibit sustained high fluxes associated with an acceleration event (e.g., Desai & Giacalone, 2016; Mitchell et al., 2020), the accelerated populations and whistler‐mode emissions are very temporally narrow. Proton fluxes at any given energy are depleted within less than a minute of their arrival to and observation by Juno. This suggests quasi‐static structures and wave‐particle coupling occur in a more pulsed, transient manner reminiscent of ∼5 min transient conics observed at Saturn (Mitchell et al., 2009). The very short duration of these dispersive conics throughout the period we observe ∼1 keV core protons indicates acceleration/heating configurations are rapidly generated and dissipated. The exact nature of how these structures form and subsequently dissipate, as well as their relation to the existing charged particle populations on these field lines remain open and interesting questions for future analyses.

Finally, while the core population's fluxes peak at similar energies to the plasma observed in Jupiter's magnetosheath (McComas, Szalay, et al., 2017), the plasma composition is markedly different than magnetosheath plasma. Instead, the compositions and energies observed during the dispersive conic events are remarkably similar to Jupiter's magnetotail at similar midnight local times. Specifically, heavy ions corresponding to various charge states of oxygen and sulfur are observed above a few keV/Q. As these heavy ions originate from Io, there is strong compositional evidence the observed plasmas during the period discussed in this study are magnetospheric in origin. This is also consistent with Io‐genic heavy ions accelerated toward Jupiter's polar cap through large potentials (Clark, Mauk, Kollmann, Paranicas, et al., 2020). Additionally, the core protons are nearly isotropic, apart from the anti‐Jupiterward depletion, which also indicates Juno is on closed magnetic field lines loaded with Jovian magnetospheric plasma. Therefore, these measurements provide observational evidence of closed field lines mapping to some of Jupiter's most polar regions. Jupiter's polar magnetospheric topology is fundamentally different from Earth's due to the internal plasma source Io at Jupiter (McComas & Bagenal, 2007) and modeling efforts have suggested Jupiter's polar cap is predominantly closed (Zhang et al., 2021). While the observations here for a single instance do not systematically prove Jupiter's polar magnetosphere is topologically closed, they do provide a concrete example where Jupiter's extreme high latitude polar magnetosphere is closed. As these events occur near midnight local time, likely map to large distances in the magnetotail (Zhang et al., 2021), and exhibit very similar compositional and energy spectra to those in the magnetotail, these types of events can seed energetic particles into Jupiter's magnetotail.

### 4.1 Summarizing Our Key Findings

1. Two distinct populations of protons are observed connected to Jupiter's polar‐most regions: a ∼1 keV core and transient ∼1–300 keV dispersive conic population
2. Contemporaneous whistler‐mode emissions indicate the existence of anti‐planetward field‐aligned electron beams
3. The dispersive population is likely perpendicularly accelerated within a planetocentric distance of ∼3–5 R_J_
4. Of the possible source mechanisms, we rule out quasi‐static and reconnection‐driven acceleration, and suggest wave‐particle heating in a pressure‐cooker setup is responsible
5. These acceleration structures form and subsequently dissipate rapidly ∼10's seconds
6. Magnetospheric heavy ions are observed simultaneously with the two proton populations, providing evidence Jupiter's polar‐most field lines can be topologically closed
7. This mechanism injects energetic particles into Jupiter's magnetotail

## Data Availability

The JNO‐J/SW‐JAD‐3‐CALIBRATED‐V1.0 data set (V04) can be obtained from the Planetary Data System (PDS) at https://pds-ppi.igpp.ucla.edu/mission/JUNO/JNO/JAD, JEDI data can be obtained at https://pds-ppi.igpp.ucla.edu/mission/JUNO/JNO/JEDI, and Waves survey can be obtained at https://doi.org/10.17189/1519710.
